# Surgery and the General Practitioner

**Published:** 1908-02-29

**Authors:** 


### Editor's Letter-Box.

[Our correspondents are reminded that prolixity is a great bar to publication, and that brevity of style and conciseness of statement greatly facilitate early insertion.]

### "SURGERY AND THE GENERAL PRACTITIONER."

A Reply by a General Practitioner.

In the article on the above, in The Hospital of February 22, exception is taken to the alleged want of courage, or of energy, on the part of the general practitioner in general, to undertake surgical operations: (1) In the case of his better-class patients, because of possible loss of prestige and practice in the event of not meeting with success; (2) in the case of his poorer patients, on account of inconvenience and trouble to himself. Now there are other reasons more cogent than these which will explain better this seeming reluctance. To begin with, it may fairly be stated that as a rule the general practitioner *does* like to undertake his own cases (and others, too, if he can get them). He is not chary of operating from any sense of fear arising from want of faith in his own skill. He does not lack courage. We do not infer that *all* are alike in this. Some men prefer medicine to surgery and therefore have no desire to operate; others, though not a large class fortunately, have no real love for their profession, let alone any particular branch of it; such as these do not, of course, come into our category. But, taken as a whole, general practitioners would prefer to do their own surgical operations and not have the practice thereof *taken* out of their hands, as is so often the case at present. Let us consider first the better class of patients. Now what happens? The general practitioner in a country district is a man well received by his patients, beloved by them, and of his skill they profess to have no doubt. But let occasion arise for any serious operation, the first thing required, of course, is a consultation, and generally some "big gun" is sent for. He gives his opinion, let us say, in favour of an operation. Well, who is to perform it? Is it not a fact that in 99 cases out of 100 the friends desire him to undertake it? The willingness or the skill of the usual medical man does not count; they do not even enter into consideration. One has to bear in mind that the friends, impressed by the gravity of the case, require "eminent counsel," first because they have a feeling they must leave no stone unturned that will help, and secondly because they are influenced in no small measure by public opinion. We do not put this in a satirical sense. One implies only that *nolens volens* the human being, by the very nature of his surroundings, must be influenced more or less by what others will say or think. But more often than not the medical attendant himself asks for a second opinion, and if a specialist is called in the result is just the same—it is a sort of foregone conclusion that he will operate. And the same factors are at work in other and less serious cases, where the doctor sees no necessity for a second opinion and is quite ready to undertake the operation himself. The mere mention of "operation" suffices, and we find him compelled by force of circumstances to give way on account of the same love of publicity and, we may add, of self-importance. The calling-in (as it is expressed) of another man seems to lend a sense of importance to the cases and attracts attention. We do not mean to infer by this that everyone is actuated by such motives (nor that any are wholly controlled by them), but in very many cases it is so, though it must be granted that the predominant idea is "to do the best they can for the patient." Thus the general practitioner has no control in the matter; he cannot be arbitrary and insist, he can only acquiesce in such tactful manner as not to betray his own desire or regret, and so his dignity remains untouched. In such instances, therefore, it is not his fault that a specialist is called in to do his work, he is neither chary of doing it, nor is he fearful—he must bow to the inevitable.

Next, as regards his poorer patients. We certainly must object to the expressions applied to us concerning "inconvenience and trouble," "content to send to nearest clinics," "perfunctory manner of operating," "sublime disregard of asepsis." They convey a general condemnation of our work. But beyond this we do not propose to enter into any defence of our character. The work of a country surgeon is well known; his devotion to and love for it is so thoroughly appreciated as to make it unnecessary. Probably the writer is only "using the spur" for a supposed "flagging horse." Now one of the *chief* reasons for not attempting operations amongst our poorer patients is a very grave *regard* for asepsis, and because the surgeon does not wish to do his work perfunctorily. How is it possible, in a small dark room, probably in a cottage, not by any means noted for absence of dirt and dust, crowded up as so many cottages are with useless ornaments, etc., how is it possible to undertake an operation or to do properly anything requiring asepsis and care? Occasionally, of course, circumstances call for urgent attention and one has to act as best one can, but no one would voluntarily choose such an "operating theatre" either for his patient or himself. In writing this, we recall our own experience in a small country town. Ten years ago a cottage hospital was erected, since then we have taken in for operation not only our town cases, but also those of the surrounding district. We have shirked no one on account of trouble, and have taken everything that turned up—small or great. *Before* then we never thought of doing any operation, all were sent away, simply and solely for the reasons stated above. And we feel sure this is the general feeling amongst general practitioners who love their work; they are *willing* enough to undertake whatever is required of them, but it must be under such conditions as will give them satisfaction in result and insure the safety of their patients. A. C. H.

[Our leading article was in no sense an attack on the "operative skill" of the general practitioner, as our correspondent imagines. Nor did we lament in it the negligence of the practitioner to undertake serious major operations that call for expert opinion and a specialist's help. But it is unfortunately true that the average practitioner does *not* pay the attention which he ought to minor surgery. There are exceptions, of course, and many of them—would that their examples might stimulate the rest! The whole article was an appeal to practitioners to seize such opportunities for practice which would tend to make them operate "under such conditions as will give them satisfaction in result and insure the safety of their patients."—Ed. The Hospital.]

